# State of the Art in 2022 PET/CT in Breast Cancer: A Review

**DOI:** 10.3390/jcm12030968

**Published:** 2023-01-27

**Authors:** Jules Zhang-Yin

**Affiliations:** Department of Nuclear Medicine, Clinique Sud Luxembourg, Vivalia, B-6700 Arlon, Belgium; juleszhangyin@gmail.com

**Keywords:** breast cancer, PET/CT, FDG, FES

## Abstract

Molecular imaging with positron emission tomography is a powerful and well-established tool in breast cancer management. In this review, we aim to address the current place of the main PET radiopharmaceuticals in breast cancer care and offer perspectives on potential future radiopharmaceutical and technological advancements. A special focus is given to the following: the role of ^18^F-fluorodeoxyglucose positron emission tomography in the clinical management of breast cancer patients, especially during staging; detection of recurrence and evaluation of treatment response; the role of 16α-^18^Ffluoro-17β-oestradiol positron emission tomography in oestrogen receptors positive breast cancer; the promising radiopharmaceuticals, such as ^89^Zr-trastuzumab and ^68^Ga- or ^18^F-labeled fibroblast activation protein inhibitor; and the application of artificial intelligence.

## 1. Introduction

Breast cancer (BC) is the most frequent cancer diagnosed in women and accounts for about one in eight women diagnosed with cancer worldwide [1]. The vast majority of BC occurs in women over 50 years of age. Although BC can occur in men, the incidence is very low. In the United States, there will be an estimated 287,850 new cases of invasive BC in 2022 [2]. Furthermore, 43,250 deaths are expected to be recorded from women with BC [2]. It is the second-leading cause of cancer-related death worldwide after lung cancer [1].

Several treatment options (surgery, radiotherapy, chemotherapy, targeted therapy, endocrine therapy) are available in BC management. Treatment is tailored to the biological and histological characteristics of the tumor and to the stage of the disease. Accurate staging, restaging, and response evaluation are essential for planning further optimal management [3].

In the field of oncology, molecular imaging, e.g., positron emission tomography (PET), is commonly used in cancer management, especially for staging purposes. PET can provide quantitative pictures of biological processes even at a low radiative dose. Moreover, PET has proven to have a better spatial resolution, high sensitivity, and regional tracer uptake quantification than other molecular imaging techniques. Since early 2000, PET has always been performed with computed tomography (CT) in cancer imaging.

This article aims to provide a state-of-the-art picture of the current clinical applications of PET/CT in BC, particularly for ^18^F-fluorodeoxyglucose (FDG) PET/CT and 16α-^18^Ffluoro-17β -oestradiol (FES) PET/CT, and also to discuss innovative radio-pharmaceuticals, the application of artificial intelligence (AI) in BC, and cost-effectiveness considerations.

## 2. FDG PET/CT

### 2.1. At Initial Staging

In its BC staging, the American Joint Committee on Cancer (AJCC) uses a set of T, N, and M criteria [4]. Imaging is an essential tool in the staging of BC. BC should be staged because it provides meaningful information to guide treatment and, if possible, predicts patients’ prognosis [5]. Indeed, there is a five-year survival rate of 76% to 99% for patients with locoregional BC compared to those with distant metastases, with a five-year survival rate of 20% to 28% [2]. FDG PET/CT could help accurately stage the disease.

#### 2.1.1. Initial Detection of Primary Breast Tumor

When compared to breast magnetic resonance imaging (MRI), ultrasound, and mammography during the evaluation of lesions in primary breast tumors, FDG PET/CT has a lower sensitivity [5], especially for lesions less than 1 cm, due to the partial volume effect and limited spatial resolution [3]. Moreover, compared to the common ductal breast cancers, other histologic cancers, e.g., lobular breast cancers, tend to exhibit low FDG avidity, which may be invisible at FDG PET [6].

Furthermore, apart from low sensitivity, FDG PET/CT has poor specificity within the breasts [7]. Whole-body FDG PET/CT is not valuable because of its low specificity and sensitivity in the initial detection of primary breast tumors [4,8]. Compared to PET, breast MRI has greater sensitivity and accuracy in detection, in delineating primary tumor volume, and in assessing multifocality, and it’s the mainstay [3,9,10,11].

#### 2.1.2. Locoregional Nodal Metastases

Determining the difference between extra-axillary and axillary node involvement is clinically relevant for evaluation of locoregional nodal metastases. Lymph node biopsy is commonly used for axillary nodal staging [4]. Indocyanine green or blue dye techniques can identify the sentinel node in at least 97% of breast cancer patients [12]. In the remaining axilla, the sentinel node highly predicts the status of the disease [13,14]. Regarding auxiliary nodal metastases, FDG PET/CT has low sensitivity [15] because the axillary nodal metastases are usually small. In a meta-analysis of 19 studies, including 1729 patients, the sensitivity of PET to detect axillary involvement was only 66% [16].

This same meta-analysis highlighted a better specificity of FDG PET/CT for axillary nodes; it was 93% [16]. FDG PET/CT is preferred for its specificity for axillary nodes rather than its sensitivity [17,18]. Therefore, an FDG-positive axillary node could be an indication of nodal malignancy, but other factors that cause false-positive FDG avidity should also be considered [3], such as COVID vaccination [19].

Sentinel node evaluation rarely identifies locoregional extraaxillary nodes and lymph node dissection for BC is usually limited to levels I and II of the axillae. This is where FDG PET/CT becomes valuable, as it can detect unsuspected extraaxillary nodal metastases, such as in infraclavicular and supraclavicular areas or the internal mammary chain. It may have important implications in the management of surgery [20] and radiotherapy [21,22]. Thus, at initial staging, the detection of extraaxillary nodal metastases by FDG PET/CT affects the prognosis and the patient’s stage [3].

#### 2.1.3. Initial Detection of Distant Metastases

In distant metastases, the commonly used imaging techniques in patients with BC are bone scintigraphy and thorax, abdomen, and pelvis (TAP) CT [3]. FDG PET/CT has recently been put into practice, and it can detect distant metastases that are unsuspected in locally advanced BC, such as in distant nodes, as well as pleural, hepatic, splenic, adrenal, and pelvic metastases [23,24,25]. Indeed, in the study by Groheux et al. the authors showed that FDG PET/CT had a sensitivity and specificity of 100% and 99.1%, respectively, for the diagnosis of pleural metastases (vs. 50% and 100% for the CT). Moreover, PET/CT detected supra-diaphragmatic distant lymph nodes in 18 patients and infra-diaphragmatic nodes in 4 patients [23].

In the study by Fuster et al. including 60 patients, PET/CT outperformed, certainly compared to conventional imaging, in detecting distant metastases, with sensitivity and specificity of 100% and 98%, respectively, compared to 60% and 83%, respectively, for conventional imaging [24].

In another study including almost twice as many patients (103), PET/CT had similar sensitivity and specificity of 100% and 95%, allowing detection of distant metastases in nearly a quarter of the patients (23%) [25].

FDG PET/CT is more sensitive and specific than CT or bone scintigraphy for detecting lytic or mixed bone metastases or bone marrow involvement [26,27].

**Figure 1:**
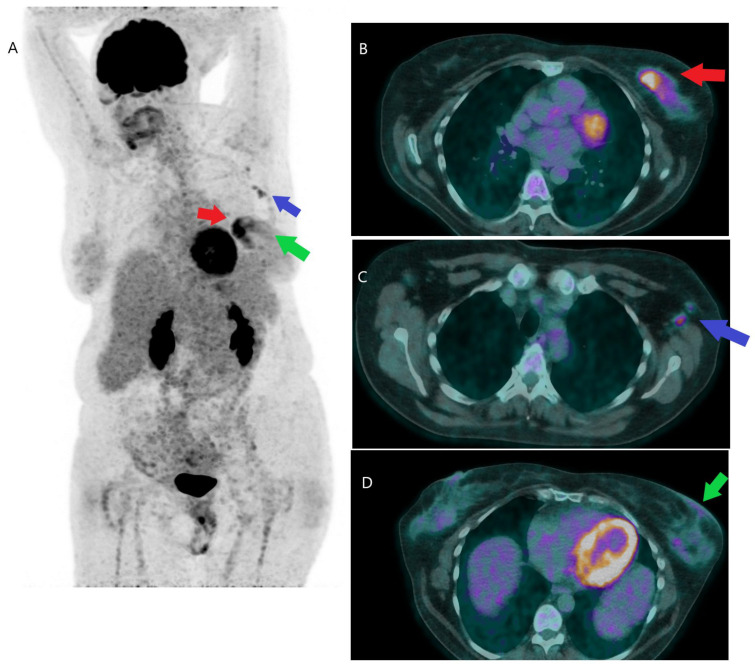
FDG PET/CT in initial staging. A 52-year-old woman with a newly diagnosed inflammatory left breast invasive ductal carcinoma of 29 mm, was referred for initial staging with FDGPET/CT. The PET/CT MIP image (**A**) shows the primary cancer (red arrow), axillary adenopathies (violet arrow), and cutaneous involvement (green arrow). Image (**B**) (fusion image, trans-axial view) shows the primary lesion of the left breast (red arrow). Image (**C**) (fusion image, trans-axial view) shows the axillary lymph node involvement of Berg’s level I (violet arrow). Image (**D**) (fusion image, trans-axial view) shows the cutaneous involvement (green arrow).

**Figure 2:**
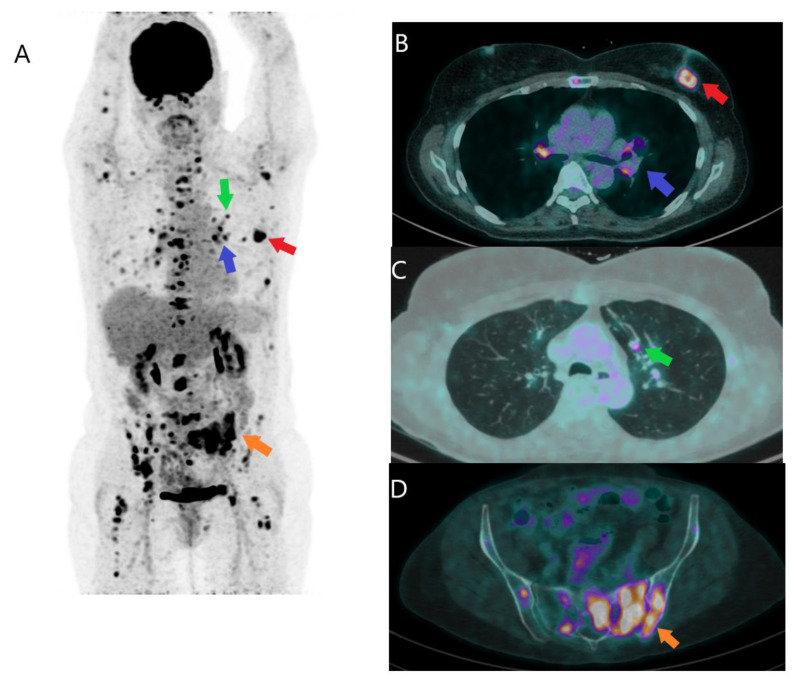
FDG PET/CT in initial staging. A 41-year-old woman with a newly diagnosed triple negative left breast invasive ductal carcinoma of 21 mm, was referred for initial staging with FDG PET/CT. The PET/CT MIP image (**A**) shows the primary cancer (red arrow), mediastinal and hilar adenopathies (violet arrow), lung metastases (green arrow), and extensive bone involvement (orange arrow). Image (**B**) (fusion image, trans-axial view) shows the primary lesion of the left breast with necrotic center (red arrow) and the bilateral mediastinal and hilar adenopathies (violet arrow). Image (**C**) (fusion image, trans-axial view) shows a lung nodule in left upper lobe (green arrow). Image (**D**) (fusion image, trans-axial view) shows extensive bone involvement of sacrum and ilium (orange arrow).

Ultrasound, mammography, axillary nodal pathologic evaluation, and breast MRI perform well in T and N staging, compared to FDG PET/CT, which can be significant for M staging. Therefore, FDG PET/CT should be utilized for patients exhibiting high advanced disease risk, such as in stages IIB–IIIC [28] (Figure 1 and Figure 2).

Patients with a small tumor of ≤2 cm (T1 in the TNM classification) are treated with surgery combined with sentinel node evaluation. PET has a limited spatial resolution and its performance is inferior to the sentinel node technique [16]. In addition, there is almost no distant metastasis identifiable on imaging in T1N0 disease (AJCC stage I). In a multicentre study of 325 women with operable breast cancer, the FDG PET (without CT component) suggested distant metastases in 13 patients. Only 3 (0.9%) were confirmed as metastatic disease and 10 (3.0%) were false positives [29]. For patients treated for stage I cancer, extension workup may delay treatment and/or cause unnecessary anxiety. So, the use of PET in Stage I disease is definitively not recommended.

### 2.2. Recurrent Disease

Evaluating distant metastasis and locoregional recurrence is essential [30]. Even though there is a grave prognosis carried by recurrent disease, the survival rate is likely to improve if early detection of the recurrence is established. In cases of elevated serum markers, physical examination findings, or clinical symptoms suggesting malignancy recurrence, imaging is usually recommended for further evaluation. Based on the clinical guidelines of the European Society for Medical Oncology (ESMO), early detection/identification of local recurrences is the main goal during the surveillance of patients suspected of BC recurrence [31]. Nevertheless, the clinical guideline states that, in asymptomatic patients, no imaging tests can generate a survival benefit, FDG PET/CT included [31].

In contrast, National Comprehensive Cancer Network (NCCN) and ESMO clinical guidelines state that, in cases where traditional imaging techniques conflict during the identification of the site of relapse, FDG PET/CT can be beneficial [5,11]. Furthermore, FDG PET/CT can enhance the identification of isolated metastatic lesions and locoregional relapse [32,33]. The same imaging modality has been proposed when it comes to inconclusive conventional imaging and increasing tumor markers. This can be seen with FDG PET/CT, which has a high specificity of at least 69% and a high sensitivity of at least 77% [34]. These FDG PET/CT rates are higher in patients with suspected radiological recurrence and with increased serum CA15.3 levels; a sensitivity of 92.7% was recorded [34].

FDG PET/CT is essential during the assessment of recurrent disease. To compare the diagnostic accuracy of bone scan, contrast-enhanced CT, and FDG PET/CT, 100 patients with suspected recurrent BC were evaluated [35]. According to the study, FDG PET/CT had a higher diagnostic accuracy than bone scan and contrast-enhanced CT. FDG PET/CT had few false positives and negatives compared to other imaging methods. When compared with MRI, FDG PET/CT tends to exhibit at least equal accuracy in detecting the recurrence of disease [36].

A recent meta-analysis published in 2016 concerning FDG PET/CT in BC recurrence included a total of 26 studies involving 1752 patients. Among them, 56.8% were referred for elevated tumor markers, 33.9% for suspicion revealed by conventional imaging, and 9.4% for clinical symptoms. The pooled sensitivity and specificity of FDG PET/CT were 0.90 and 0.81, respectively [37].

In another meta-analysis published in 2012 including 13 studies that evaluated the performance of FDG PET/CT to detect recurrence in the presence of increased tumor markers, the authors found a sensitivity of 87.8%, a specificity of 69.3 %, and a diagnostic accuracy of 82.8% [38].

FDG PET/CT is recommended in cases of suspected recurrence and for the staging of a proven recurrence of BC.

### 2.3. Evaluation of Treatment Response

#### 2.3.1. Metastatic Disease

At CT, the size of lesions in metastatic BC helps measure treatment response. On the contrary, FDG PET can measure metabolic changes, which is better in predicting treatment response than anatomic changes [39]. Two studies discovered that FDG PET could evaluate response vs. non-response after 1–3 therapy cycles [40,41]. Moreover, other studies found that FDG PET can differentiate response from non-response, which was relevant to various and distinct chemotherapy and hormonal courses [42,43].

**Figure 3:**
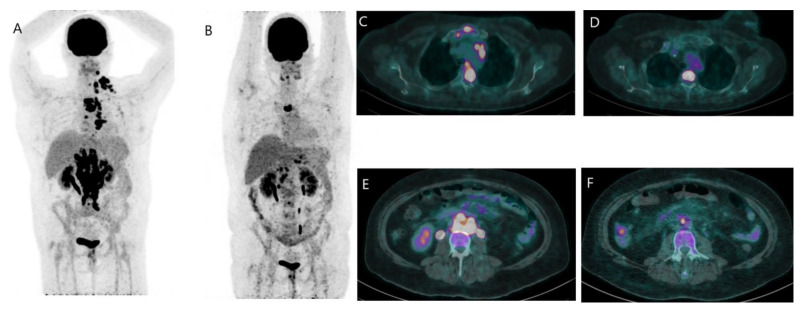
FDG PET/CT in evaluation of treatment response. A 47-year-old woman with metastatic HER2+ BR was treated by paclitaxel and pertuzumab and was referred for evaluation of treatment response. The PET/CT MIP images (**A**,**B**) show a significant partial metabolic response at 3 months of interval. Images (**C**,**D**) (fusion image, trans-axial view) show the regression of mediastinal adenopathy and sternum lesion and also the persistence of a bone lesion of T4. Images (**E**,**F**) (fusion image, trans-axial view) show the important regression of retroperitoneal lymph node involvement.

**Figure 4:**
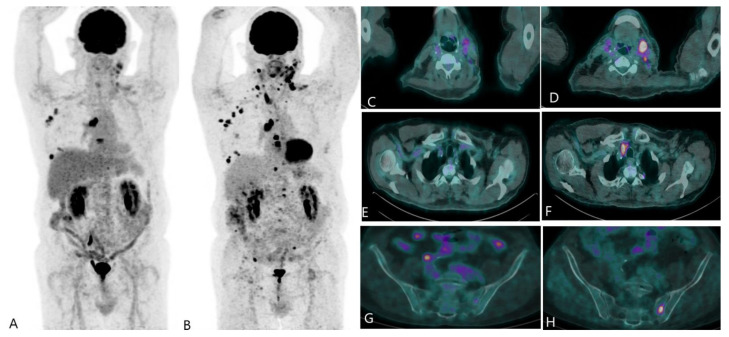
FDG PET/CT in recurrence disease and in evaluation of treatment response. A 65-year-old man was diagnosed with BR five years ago and treated by surgery, chemotherapy, and radiotherapy. He was referred for elevation of tumor marker and left cervical swelling. The PET/CT MIP image (**A**) shows a recurrence of disease, notably at left cervical, right axillary, and mediastinal lymph nodes, also a bone involvement at T7 and right ribs. The patient was treated then by capecitabine and the evaluation of treatment response was performed 5 months later. The PET/CT MIP image (**B**) of evaluation showed a progressive disease. Images (**C**,**D**) (fusion image, trans-axial view) show the morpho-metabolic progression of cervical lymph nodes. Images (**E**,**F**) (fusion image, trans-axial view) show the apparition of a superior paratracheal lymph node involvement. Images (**G**,**H**) (fusion image, trans-axial view) show the intensification of uptake of a bone lesion in the left sacrum.

Studies on the FDG PET/CT showed that it was more efficient in detecting skeletal metastases earlier than CT [44,45]. FDG PET/CT detects osseous metastases earlier when compared to CT. CT can be inaccurate during the assessment of treatment response due to the sclerotic lesions that appear at CT after treatment. These lesions may indicate osseous healing rather than a new metastasis [46,47]. Even a bone scan can be subjected to an inaccurate assessment of osseous lesion treatment response due to the increasing avidity of the bone scan that may indicate increased osteoblastic response or increased osseous malignancy during the bone healing process [47,48] (Figure 3 and Figure 4).

The PET data based on SUV changes allowed a better prediction of response to treatment compared to conventional imaging. Indeed, in the study conducted by Schwarz et al. [49], the authors compared 26 BC metastatic lesions using FDG PET vs conventional imaging, including contrast-enhanced CT. They found that FDG SUV changes in tumor were statistically significantly different between responding and nonresponding metastatic lesions. In another study by Cachin et al. [50], the authors found that FDG PET concluded complete responses for 72% of cases (34 patients), whereas the conventional imaging only achieved 37% (16 patients). Moreover, the FDG PET result was the most powerful and independent predictor of survival.

#### 2.3.2. Neoadjuvant Treatment Response for Primary Tumor

Neoadjuvant chemotherapy (NAC) was previously used to make inoperable BC resectable. It is used in down-stage disease and operable tumors to prevent axillary nodal dissection or promote breast conservation [51]. Various studies have identified a solid correlation between NAC response and early FDG standardized uptake value (SUV)_max_ changes measured during the pathological examination [52,53]. Furthermore, other studies have stated the criteria that are suitable for the prediction of NAC response in BC with varying receptor status due to the various FDG avidity measures [54,55,56,57]. On the contrary, to a lesser extent, MRI can differentiate between non-response from NAC response [51,58,59]. Nevertheless, there is no modality to differentiate total response from the partial response at the end of NAC. This is because of the persistence of low-volume residual disease despite having no sign of disease during imaging [51,60].

Various studies, however, have acknowledged the efficiency of FDG PET/CT in assessing malignant tumor treatment response, as metabolic changes can be identified earlier by FDG PET/CT semi-quantitatively [61,62,63]. In terms of prediction of outcome after NAC, FDG PET/CT is an effective tool (using PERCIST 1.0) and was a superior predictor of progression-free and disease-specific survival than RECIST 1.1 using CT [61].

### 2.4. Drawbacks of FDG PET/CT

Various factors can affect the uptake of FDG PET in BC lesions. To be precise, glucose metabolism directly relates to BC aggressiveness. In the same case, the higher ^18^F-FDG uptake in PET/CT is correlated with undifferentiated histopathology, triple negative receptorial pattern, invasive ductal carcinoma type, and elevated Ki-67 [39]. Similarly, in some conditions with low FDG uptake, there are false-negative PET/CT results. Such conditions include small lesions, low Ki-67 expression, and histology of invasive lobular carcinoma [6,64,65]. Lastly, the factors that may contribute to low PET/CT specificity in patients with high levels of tumor markers include reconstruction artifacts, degenerative bone disease, breast expansion, and lung inflammation [38].

## 3. FES PET/CT

**Figure 5:**
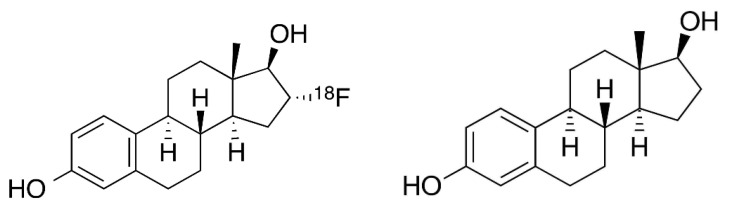
Comparison of chemical structures of FES (left-hand side) and of oestradiol (right-hand side).

FES is an analogue of oestradiol fluorinated on the carbon 16 of the D ring (Figure 5). Various fluorinated oestradiol analogues have been proposed during the last three decades for clinical PET imaging. Among them, the FES seems to be the most effective, as it can be produced with a high specific activity and it has a good binding affinity to the oestrogen receptors (ERs) [66,67,68]. FES uptake depends on both ER density and the availability of ER for ligand binding [69]. Thus, a negative FES PET does not mean there is no malignancy [70].

Among patients with BC, 70% have ER-positive tumours [66]. Indeed, BC progresses through increased transcriptional activity due to over-expressed ERs. The ER status is also a major prognostic indicator, as it is considered to be the primary predictor of the response to endocrine therapy [71]. The therapeutic strategies of ER+ BC include, on one hand, the reduction of circulating ovarian oestrogens or of peripherally produced oestrogen (in postmenopausal women) with aromatase inhibitors, and on the other hand, the application of selective ER modulators for receptor blockade by tamoxifen, which is a selective ER modulator having both ER agonist and antagonist properties, working primarily as antagonists in tumors, or by fulvestrant, which is a selective ER down-regulator and a pure ER antagonist that accelerates the degradation of the ER. Moreover, in addition to the approximately 20–30% of women with clinically ER- breast cancers who will not respond to endocrine agents due to the lack of a therapeutic target, 25–50% of initially ER+ breast cancers also exhibit de novo resistance to anti-oestrogens; this proportion is even more important in patients after previous failure of endocrine treatment [72,73].

FES PET/CT can play a complementary role for FDG PET/CT, as the latter can be negative in some histology types, such as the lobular type or less aggressive forms [6], and thus improve the sensitivity of PET imaging, especially for the detection of those tumours, in particular, of a small size, and also for its specificity to differentiate inflammatory and infectious lesions that are usually FDG-positive [71].

Sensitive detection of cancer tissue is important for staging or restaging, but the assessment of the over-expression of hormone receptors is important for the management of the patient. In cases of metastatic spread, FES PET can depict, non-invasively and at a whole-body level, those lesions that are ER-positive and likely to respond to hormone therapy.

In the past two decades, various studies have looked into FES PET in BC and shown its efficiency in assessing and predicting early response to endocrine therapy. Moreover, its efficiency being a biomarker of functional ER expression was also discovered. While under the trade name EstroTep^®^, FES was finally approved in 2016 in France. It was to be used clinically for patients with initially recurrent ER-positive breast cancer where biopsy is impossible [74].

In the US, FES, under the trade name Cerianna^®^, was approved in 2020 to be used as an adjunct to the biopsy in patients having metastatic/recurrent ER-positive breast cancer [75,76]. FES PET/CT evaluates the functional ER expression of the whole body in a non-invasive manner, thus making it an ER expression marker [66]. Therefore, FES PET/CT is more accurate in assessing ER-positive disease, detecting and identifying heterogeneity present at the site of infection, and assessing therapy response [74].

Recent studies show that FES PET/CT can efficiently categorize temporal and spatial disease heterogeneity. Temporally, in this case, refers to the same disease sites on serial scans, while spatially refers to various disease sites at one point [77,78]. According to Linden et al. FES PET/CT effectively assesses ER blockade by various endocrine therapies: FES tumor uptake was decreased to a greater extent by estrogen receptor blocking agents, i.e., fulvestrant and tamoxifen, than estrogen concentration-lowering agents—the aromatase inhibitors [79].

**Figure 6:**
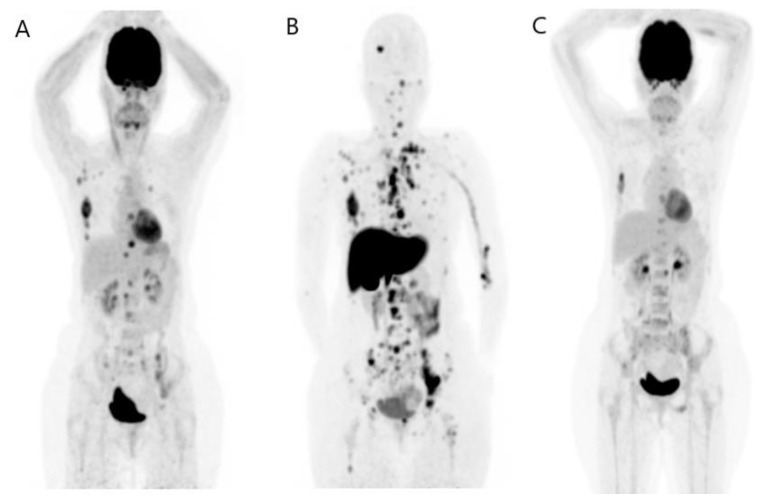
FES PET/CT in treatment guidance. A 50-year-old woman was diagnosed with BR eight years ago and treated with surgery, chemotherapy, and radiotherapy. A recurrence was detected by FDG PET/CT (**A** for MIP image). As the tumor was initially RH+, treatment with fulvestrant was considered. A FES PET/CT was performed prior. Its MIP image (**B**) shows all lesions FDG+ were FES+ and it detected other FDG- lesions. At the evaluation of fulvestrant’s treatment response 3 months later, the FDG PET/CT (**C** for MIP image) shows an important partial metabolic response.

**Figure 7:**
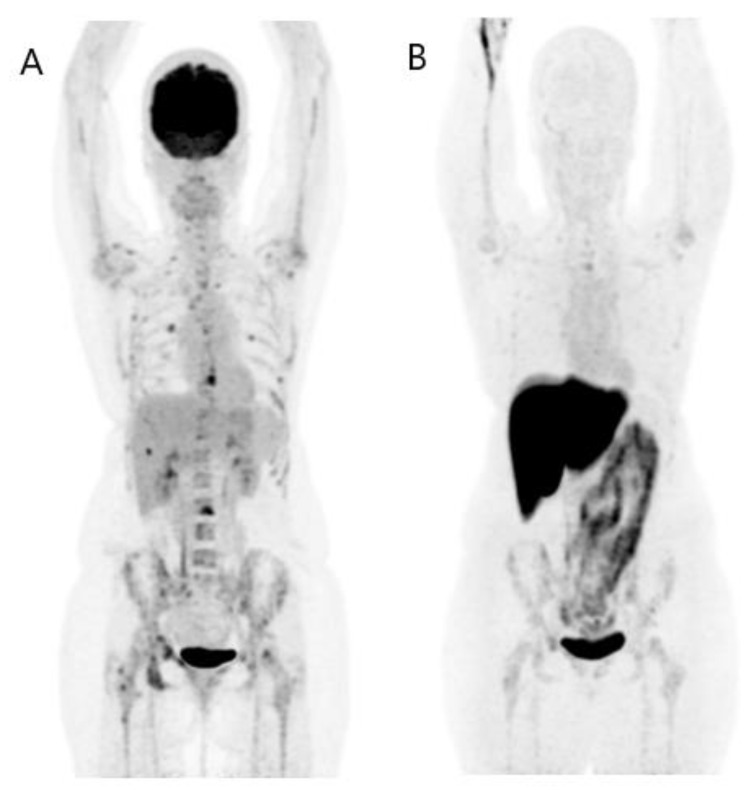
FES PET/CT in treatment guidance. A 48-year-old woman was diagnosed with bifocal BR 12 years ago and treated with surgery, chemotherapy, and radiotherapy. In the primary breast tumor, there were two contingents: one was HER2- RH- and another was HER- RH+. A recurrence was detected by FDG PET/CT (**A** for MIP image). A FES PET/CT was also performed (**B** for MIP image) and was negative. Finally, treatment with eribuline was preferred to palbociclib–fulvestrant because the latter would probably be ineffective.

To summarize, in adult patients with BC initially expressing ERs, FES PET/CT is useful in the following clinical settings: (Figure 6 and Figure 7)

- Characterization of known or suspected metastatic lesions as expressing ERs; and
- Treatment guidance and monitoring.

## 4. Other Radio-Pharmaceuticals

### 4.1. ^89^Zr-Trastuzumab

In BC, the outcome of human epidermal growth factor receptor (HER)2-positive metastatic disease has fundamentally improved since the development of effective HER2 targeting agents, such as trastuzumab, pertuzumab, and trastuzumab–emtansine. HER2 status can be discordant between primary and residual or metastatic lesions, and HER2 expression can be heterogeneous [80].

Trastuzumab, a monoclonal antibody, is labeled with ^89^Zr for identification of BC cells that over-express the Her2 receptor, as in the case of HER2+ subtypes of BC and luminal B. ^89^Zr-trastuzumab is clinically used in the identification of HER2+ BC lesions. It also identifies metastasis and positive lymph nodes [81].

In a prospective study including 34 HER2+ and 16 HER2− BC patients, an SUV_max_ cutoff of 3.2 in ^89^Zr-trastuzumab PET/CT showed a sensitivity of 76% and specificity of 62% to distinguish HER2+ from HER2− lesions [82].

A study led by Bensch et al. compared the ^89^Zr-trastuzumab uptake in HER2+ and HER2- cases using ^89^Zr-trastuzumab PET/CT. In the findings, there was no detection of ^89^Zr-trastuzumab in HER2-, while in HER2+, there was detection of ^89^Zr-trastuzumab. Therefore, since tumor heterogeneity can cause changes in BC molecular features during treatment, ^89^Zr-trastuzumab PET is essential in unresponsive cases to evaluate the state of HER2 amplification. Moreover, ^89^Zr-trastuzumab PET/ CT was performed in 20 patients with HER2-negative primary cancer and in 3 patients, increased uptake of ^89^Zr-tratsuzumab was found in metastases, indicating a change in HER2 expression between primary and metastatic lesions, allowing the anti-HER2 therapy adjustment [83].

Up to date, there is no data available concerning the prognostic value of ^89^Zr-trastuzumab PET/CT, but its value in predicting therapy response of a HER2-targeting antibody–drug conjugate was assessed in the ZEPHIR trial. In this study, conducted by Gebhart et al., including 56 HER2+ metastatic BC patients, baseline ^89^Zr-trastuzumab PET/CT indicated that patients with ^89^Zr-trastuzumab uptake were associated with longer trastuzumab emtansine treatment duration, compared to those with no uptake (11.2 versus 3.5 months) and allowed to select patients who will benefit from HER2-targeting antibody–drug conjugate trastuzumab emtansine [84].

### 4.2. ^68^Ga- and ^18^F-Labeled Fibroblast Activation Protein Inhibitor (FAPI)

Fibroblast activation protein (FAP) is highly expressed in the stroma of a variety of human cancers and is therefore considered a promising target structure for diagnostic and therapeutic approaches. It is an endopeptidase that is mainly involved in matrix remodeling and intracellular signaling regulation [85]. The FAP is known to be overexpressed by cancer-associated fibroblasts and thus upregulated in many neoplastic conditions [86]. In oncology, almost all carcinomas and sarcomas are FAP-positive [87]. On the contrary, FAB is virtually absent in healthy tissue, while present in processes with tissue remodeling. Thus, it is a valuable biomarker for a multitude of cancers. BC is also characterized by a strong desmoplastic reaction [88].

^68^Ga- fibroblast activation protein inhibitor (FAPI) targets tumor stromal and FAP visualization. ^68^Ga-FAPI is known for its high tumor-to-background ratio and fast renal clearance. With these features, ^68^Ga-FAPI is ideal for multiple types of tumors. Kratochwil et al., in a retrospective study, showed ^68^Ga-FAPI PET/CT had uptake in 28 different kinds of cancer, including BC [89]. The study lead by Halil Komek et al. showed that ^68^Ga-FAPI is highly sensitive in identifying the primary tumor in BC and high SUV_max_ [90].

The new radiolabeled FAPI, ^18^F-FAPI, is a specific tracer used during cancer imaging. According to the study by Hu et al., ^18^F-FAPI is highly effective in detecting lesions in patients with BC. It is an efficient tracer during the imaging of malignant tumors. The study also showed that, in terms of biodistribution, ^18^F-FAPI had high tumor-to-background ratio [91]. Therefore, ^18^F-FAPI is an excellent alternative to ^68^Ga-FAPI.

### 4.3. Theranostics Application

The expression of somatostatin receptor was found in 21–46% of BC specimens using in vitro autoradiography [92]. Somatostatin receptor antagonists can be promising candidates for BC theranostics [93]. Currently, there is an ongoing phase II clinical trial investigating ^177^Lu-DOTATATE for the treatment of stage IV or recurrent BC. This American study includes 10 patients. The patients receive ^177^Lu-DOTATATE IV over 30–40 min during weeks 1, 8, 16, and 24 in the absence of disease progression or unacceptable toxicity. The primary outcome is to assess the objective response rate. The authors also aim to evaluate the incidence of adverse events as a secondary outcome [94]. The results could be interesting for further theranostics application in BC.

## 5. Artificial Intelligence

In medical imaging, AI can be used in image processing, medical database retrieval, artificial neural networks (ANN) to classify images automatically, and computer vision. Moreover, AI can improve the accuracy of abnormality detection, dosage calculations, and interpretation of findings [95].

AI can be helpful in pathology prediction, detection, predicting early metastatic disease, survival estimation, and post-therapeutic evaluation [96]. Furthermore, AI can enhance ^18^F-FDG PET imaging quality by improving the attenuated correction even with structural imaging [96].

However, AI in PET has its limitations. First, AI requires massive amounts of interpreted data for development and learning, making it less reliable for small datasets. Secondly, the reliability and reproducibility of AI algorithms are needed. As modeling continues to be complicated, the AI’s ‘black box’ nature makes the results of various AI models challenging to comprehend and explain. At the moment, trusted healthcare AI prefers the stability and explainability of unknown and diverse data [97].

Generally, despite the disadvantages mentioned above, AI is important in PET, including the administration and synthesis of drugs, the management of patient information, the interpretation of reports, and image processing and acquisition. Moreover, AI can help researchers carry out investigations and identify novel molecular biomarkers [98].

In the BC field, there are emerging studies showing potentially interesting clinical applications. Li et al. studied the impact of using AI-enhanced FDG PET/CT in diagnosing axillary lymph node metastases, and they concluded that even if the diagnostic performance of AI was not better than that of clinicians, taking AI diagnoses into consideration may positively impact the overall diagnostic accuracy [99].

Takahashi et al., in order to increase the diagnostic accuracy of PET/CT, developed deep learning models using images derived from four different degrees of PET maximum-intensity projection. The authors obtained a promising sensitivity (80% to 98%) and specificity (76% to 92%) and suggested that a deep learning model may be able to assist nuclear medicine physicians in their diagnostic work in the future [100].

## 6. Cost-Effectiveness Considerations

The relatively high cost of FDG PET/CT in BC patients’ management can be a concern for the healthcare provider [101]. Some studies have investigated the cost-effectiveness aspects and found FDG PET/CT could have a positive impact. In a pilot study, Jager et al. found that FDG PET/CT for staging of high-risk BC reduced the number of further examinations in 42% of the patients. For stages II–III BC, using FDG PET/CT as the screening modality for the detection of distant metastases may result in incremental quality-adjusted life year gains and FDG PET/CT was cost-effective in the US and Netherlands [102]. In another US multicenter study including 799 stage II–III BC patients, the authors found FDG PET/CT may be cost-effective and even be cost-saving at one institution [103]. These findings support the continued use of FDG PET/CT in BC patients’ management.

## 7. Conclusions

BC is a significant health problem in modern society that affects women and has a high mortality rate. In this field, molecular imaging, especially PET, is commonly used to diagnose the disease. The FDG PET/CT provides valuable information through early detection, characterization of disease burden, and assessing response to treatment.

On the contrary, the advance on non-invasive techniques for molecular imaging of hormone receptors in BC, as illustrated by FES PET/CT, is more accurate for assessing ER-positive disease, detecting heterogeneity in tumors, and assessing therapy response.

Other radiopharmaceuticals have also been proven effective in identifying and differentiating BC cells. For example, ^89^Zr-trastuzumab PET is essential in unresponsive cases to evaluate the state of HER2 amplification, while ^68^Ga-FAPI is highly sensitive for identifying the primary tumor in breast cancer. Additionally, future developments such as AI and theranostics would enhance the quality of PET imaging and patient outcomes in BC.

## Data Availability

All data are available on request from the corresponding author.

## Abbreviations

ANN	artificial neural networks
AJCC	American Joint Committee on Cancer
BC	breast cancer
CT	computed tomography
ER	Estrogen Receptor
ESMO	European Society for Medical Oncology
FDG	^18^F-fluorodeoxyglucose
FES	16α-^18^Ffluoro-17β-oestradiol
FAPI	fibroblast activation protein inhibitor
HER	human epidermal growth factor receptor
MRI	magnetic resonance imaging
NCCN	National Comprehensive Cancer Network
NAC	neoadjuvant chemotherapy
PET	positron emission tomography
SUV	standardized uptake value
TAP	thorax, abdomen, and pelvis
